# Photoreceptor Degeneration Accompanies Vascular Changes in a Zebrafish Model of Diabetic Retinopathy

**DOI:** 10.1167/iovs.61.2.43

**Published:** 2020-02-27

**Authors:** Zaheer Ali, Jingjing Zang, Neil Lagali, Nicole Schmitner, Willi Salvenmoser, Anthony Mukwaya, Stephan C F. Neuhauss, Lasse D. Jensen, Robin A. Kimmel

**Affiliations:** 1 Department of Medical and Health Sciences, Linköping University, Linköping, Sweden; 2 Institute of Molecular Life Sciences, University of Zurich, Zurich, Switzerland; 3 Department of Clinical and Experimental Medicine, Linköping University, Linköping, Sweden; 4 Institute of Molecular Biology/Center for Molecular Biosciences Innsbruck, University of Innsbruck, Innsbruck, Austria; 5 Institute of Zoology/Center for Molecular Biosciences Innsbruck, University of Innsbruck, Innsbruck, Austria

**Keywords:** zebrafish, diabetic retinopathy, diabetes, pdx1

## Abstract

**Purpose:**

Diabetic retinopathy (DR) is a leading cause of vision impairment and blindness worldwide in the working-age population, and the incidence is rising. Until now it has been difficult to define initiating events and disease progression at the molecular level, as available diabetic rodent models do not present the full spectrum of neural and vascular pathologies. Zebrafish harboring a homozygous mutation in the pancreatic transcription factor *pdx1* were previously shown to display a diabetic phenotype from larval stages through adulthood. In this study, *pdx1* mutants were examined for retinal vascular and neuronal pathology to demonstrate suitability of these fish for modeling DR.

**Methods:**

Vessel morphology was examined in *pdx1* mutant and control fish expressing the *fli1a:EGFP* transgene. We further characterized vascular and retinal phenotypes in mutants and controls using immunohistochemistry, histology, and electron microscopy. Retinal function was assessed using electroretinography.

**Results:**

*Pdx1* mutants exhibit clear vascular phenotypes at 2 months of age, and disease progression, including arterial vasculopenia, capillary tortuosity, and hypersprouting, could be detected at stages extending over more than 1 year. Neural-retinal pathologies are consistent with photoreceptor dysfunction and loss, but do not progress to blindness.

**Conclusions:**

This study highlights *pdx1* mutant zebrafish as a valuable complement to rodent and other mammalian models of DR, in particular for research into the mechanistic interplay of diabetes with vascular and neuroretinal disease. They are furthermore suited for molecular studies to identify new targets for treatment of early as well as late DR.

Diabetic retinopathy (DR) is a frequent late complication of diabetes and a major cause of vision loss in working-age individuals.^1^ Prominent microvascular changes, including reduced perfusion, disrupted vessel integrity, and neovascularization, are accompanied by neural retina dysfunction and cell degeneration.^2^ Initiating vascular changes in DR involve reduced capillary blood flow, often secondary to damage and loss of endothelial cells, as well as microaneurysms.^3^^,^^4^ The consequence is insufficient retinal oxygenation and an increased risk of plasma leakage from affected vessels, which can lead to edema. These early vascular changes constitute nonproliferative DR. Later stages of disease, referred to as proliferative DR (PDR),^5^ are characterized by neovascularization with associated vessel leakiness and scarring, which can progress to irreversible vision loss.^4^

Although treatment with antiangiogenic agents often limits disease progression and improves visual acuity in patients with PDR, use of this therapeutic approach is limited by the invasive intravitreal route of administration, and by associated adverse effects, such as hypertension, infection, and global vascular perturbation.^6^ Furthermore, many patients are not responsive to antiangiogenics, or become resistant over time.^7^ Laser photocoagulation is also an option for slowing disease progression in PDR, but this cannot reverse damage or provide a permanent cure.

Neural-retinal damage, previously considered a secondary effect of vessel insufficiency, is now increasingly recognized as an early event and may in fact be a direct consequence of hyperglycemia. Subsequent vessel phenotypes may result from immune system activation triggered by cell stress and photoreceptor apoptosis.^5^  Although pathological features have been well described, the mechanistic link between hyperglycemia, and neural retinal and vascular pathologies is not well understood.^8^

There is a clear medical need to identify additional molecular regulators of disease progression in DR to provide new targets for therapeutic interventions. However, discoveries have been limited by the lack of animal models that present the full spectrum of pathological changes observed in the vasculature and neural retina of DR patients.^8^ Rodent models of hyperglycemia and diabetes, both chemically and genetically induced, typically recapitulate early vascular changes of DR but do not develop later proliferative phases. Neuroretinal changes, including photoreceptor degeneration and glial activation, are a common finding in both type 1 and type 2 rodent models of diabetes.^9^ However, rodent retinal phenotypes may not fully correlate with those of DR in humans, as nocturnal rodents are generally cone-deficient animals, whereas the human macula, responsible for visual acuity, consists predominantly of cones. Furthermore, phenotype and disease progression varies highly depending on the model used, and molecular mechanisms underlying pathogenesis and disease progression remain unclear.

The vertebrate model organism zebrafish is increasingly used to model metabolic diseases and diabetes, providing advantages of small size, and ease of maintenance and manipulation.^10^^,^^11^ Zebrafish can be rendered hyperglycemic by incubation in a high glucose solution, which can be maintained over several weeks.^11^ Ablation of beta cells also leads to a temporary hyperglycemia, which resolves owing to the regenerative capacity of beta cells in zebrafish.^10^ A longer duration diabetic state is found in zebrafish that are homozygous mutant for the pancreatic transcription factor *pdx1.*^12^ The *pdx1* mutant consistently shows decreased beta cell number, reduced insulin, and hyperglycemia from larval through adult stages. They are thus uniquely suitable among zebrafish models for studying long-term effects of hyperglycemia.

In zebrafish, retinal structure and visual system physiology closely resembles that of other vertebrates, including mammals.^13^^,^^14^ Similar to humans, which depend on the cone-rich macula for daytime vision, the zebrafish retina is adapted for daylight vision and rich in cones.^14^^,^^15^ Hyperglycemic zebrafish, caused by high glucose incubation or toxin-induced loss of beta cells, showed retinal thinning and vasculature changes, as well as disruption of cone cells.^16^^–^^18^ However, there was no evidence for neovascularization, perhaps due to limited duration of the treatments. Thus far there have been no zebrafish models for PDR, or for earlier stages of DR based on genetically induced diabetes.

Neovascularization has been seen in adult zebrafish exposed to hypoxia,^19^^,^^20^ so we hypothesized that zebrafish could show neovascularization under alternative pathological conditions, such as the persistent hyperglycemia associated with diabetes. In this study, we examined adult *pdx1* mutants for vascular and neuroretinal signs of DR and followed progression with aging. To visualize retinal vascular phenotypes, we generated zebrafish expressing the *fli1a:EGFP* endothelial cell reporter in the *pdx1* mutant background. We report here that adult *pdx1* mutant fish develop multiple vascular phenotypes similar to those observed in DR patients, including vasculopenia, vascular leakage, and late-onset retinal neovascularization. Concurrently, there is variable loss of retinal neurons that shows minimal progression over time. Overall, the diabetic *pdx1* mutant zebrafish manifests vascular and neuroretinal pathology seen in human DR, representing a promising model to clarify disease mechanisms and identify new treatment strategies.

## Methods

### Zebrafish Maintenance

Zebrafish used were previously described *pdx1* mutants,^12^ mixed males and females, maintained according to standard procedures as homozygous, heterozygous, and wild-type siblings. Studies used nontransgenics and fish containing the *fli1a:EGFP*^21^ transgene. Animals were staged by month as indicated or designated as young adult (6–9 months) middle-aged (11–18 months), or aged (>24 months). Genotyping was performed as previously described.^12^ All procedures were approved by the Austrian Bundesministerium für Wissenchaft und Forschung (GZ BMWFW-66.008/0009-WF/II/3b/2014, GZ BMWFW-66.008/0004-WF/II/3b/2014, and GZ BMWFW-66.008/0018-WF/V/3b/2017) and conducted in accordance with the ARVO Statement for the Use of Animals in Ophthalmic and Vision Research.

### Retinal Flat Mount Preparation and Imaging

Retinal or choriocapillary flat mounts were prepared as previously described.^22^ In brief, zebrafish were euthanized, and heads were fixed in 4% paraformaldehyde (PFA) at 4°C overnight. Retinae and choriocapillaries were isolated from the other ocular tissues, cut radially 3 to 4 times, and flat-mounted with the vitreal surface up on microscope slides in VectaShield mounting medium (Vector Laboratories, Inc., Burlingame, CA, USA). Vessels were visualized using an LSM700 confocal microscope (Leica Microsystems, Wetzlar, Germany). Several views were stitched together when required for visualizing the entire vasculature.

For studies of vessel leakage, zebrafish were anesthetized in 0.02% buffered tricaine, weighed, and then injected intraperitoneally with 2 to 4 µL lysine-conjugated, rhodamine-labeled dextran (70 kDa, 6.25 mg/mL) diluted in phosphate-buffered saline (PBS). After recovery in normal fish water, dextran circulated for 90 minutes before the fish were euthanized.

### Vessel Quantification and Statistical Analysis

Arterial density was quantitated in Photoshop (Adobe, San Jose, CA, USA) by calculating the vessel signal area (green pixels) divided by the total pixel area in eight areas from four individual fish per group. Vessel diameter was measured using ImageJ software (National Institutes of Health, Bethesda, MD, USA) in 15 vessels from 4 individual fish per group. Sprouts and branches were counted in 10 regions from 3 individual fish per group. Areas analyzed included dorsal, ventral, temporal, and nasal regions. Glucose transporter-1 (Glut1; Merck Millipore, Darmstadt, Germany) and zonula occludens 1 (ZO.1; Invitrogen, Carlsbad, CA, USA) staining was quantified (using Photoshop) by determining the percentage of signal overlap between regions positive for the EGFP+ endothelium and the antibody label. Transgelin1-labeled arterial vessels were outlined based on identification of weakly autofluorescent erythrocytes in the vascular lumens. Vessel perfusion was quantified (using Photoshop) by calculating the luminal dextran signal area (yellow pixels as dextran were recorded in the red channel and the vessels in the green channel) divided by the total vessel area (green pixels in the green channel). Leakage was quantified as the extra vascular dextran signal area (i.e., red pixels in dual-color overlays) divided by the total dextran signal area (i.e., red + yellow pixels in dual-color overlays).

Data were found to be normally distributed and differences between two groups were evaluated using heteroscedastic *t*-tests using Welch-correction for unequal variance, whereas multigroup comparisons were done using ANOVA with Tukey's post hoc test when comparing individual groups with equal variance. *P* values are indicated in the figures using the following symbols: *P < 0.05, **P < 0.01, and ***P < 0.001.

### Immunohistochemistry

For flat mounts, fixed eyes from adult zebrafish were washed 3 times with PBS and further incubated in PBS for 2 hours at room temperature or 24 hours at 4°C. Retinal or choroid tissues were isolated by careful dissection and incubated in proteinase K (20 µg/mL) for 5 minutes at room temperature on a rocking table. Tissues were then incubated for 30 minutes at room temperature in absolute methanol, followed by 0.3% Triton X-100 (Karl Roth, Karlsruhe, Germany) in PBS (PBS-Tx) for 30 minutes at room temperature with rocking, followed by blocking in 3% milk in PBS-Tx overnight (16–24 hours) at 4°C. After washing three times in PBS-Tx, tissues were incubated in primary antibodies recognizing Glut-1 (4 µg/mL), ZO.1 (4 µg/mL), or transgelin1^23^ (1:200 dilution) diluted in 0.3% PBS-Tx for 24 hours at 4°C. Tissues were then washed three times in PBS-Tx, incubated in PBS-Tx for 90 minutes at 4°C, reblocked in 3% milk PBS-Tx for 90 minutes at room temperature, and incubated with a secondary antibody, Alexa fluor 555, goat anti-rabbit (Life Technologies, Carlsbad, CA, USA) at a concentration of 10 µg/mL in blocking buffer, overnight at 4°C. Tissues were washed three times in PBS-Tx and then mounted within viewing chambers in VectaShield (Vector Laboratories).

Immunohistochemistry on frozen sections was performed essentially as described^24^ with the following modifications. Isolated eyes were fixed for 2 hours at room temperature in 4% PFA, followed by incubation in 30% sucrose prior to sectioning in optimal cutting temperature embedding medium (OCT). Primary antibodies (mouse anti-Zpr1, Zebrafish International Resource Center, Eugene, OR, USA; mouse anti-glutamine synthetase, MAB302, Chemicon International, Temecula, CA, USA) were diluted 1:400 in blocking solution and incubated overnight. The secondary antibody (Invitrogen) was diluted 1:1000 in PBS and incubated for 1 hour. BODIPY TR Methyl Ester (Invitrogen) was diluted 1:300 in PBS and stained for 20 minutes. Nuclei were counterstained with DAPI 1:1000 in water for 3 minutes. Fluorescence intensity of glutamine synthetase (GS) was measured in single z-slices using ImageJ software over regions spanning the inner nuclear layer (INL) to the outer limiting membrane (OLM). Cone photoreceptor length was estimated using ImageJ software (*https://fiji.sc/*).^25^ Ultraviolet (UV) cones were identified based on position and pattern of BODIPY staining of photoreceptor outer segments.^24^^,^^26^ UV cone length (outer and inner segment length) was measured in 78 control cells, 73 cells from mutant with mild electroretinogram (ERG) phenotype, and 58 with severe phenotype. Double cone length (synapse to distal edge of inner segment) was measured in 170 control cells, 103 cells from mutants with mild ERG phenotype, and 209 with severe phenotype. Graphs were prepared in Prism (GraphPad, San Diego, CA, USA).

### Histology and Electron Microscopy

For histology and transmission electron microscopy, whole eyes were fixed in a mixture of 4% PFA/2.5% glutaraldehyde diluted in 0.1M Na cacodylate (pH = 7.4) at room temperature (RT) for 3 hours followed by overnight at 4°C. Retinas were rinsed in 0.1M Na cacodylate, then incubated in 0.1M Na cacodylate containing 6% sucrose. Specimens were washed with 0.1M Na cacodylate buffer and postfixed in 2% osmium tetroxide buffered in 0.1M Na cacodylate at 4°C. After 1 hour, specimens were washed two times with 0.1M Na cacodylate buffer and three times with distilled water. Staining with 1% aqueous uranyl acetate was performed en block at 4°C for 1 hour. Specimens were then washed three times with distilled water and dehydrated in a series of ascending concentration of ethanol and propylene oxide. Infiltration took place in three steps, and finally the samples were embedded in Araldite 502/Embed 812 (Electron Microscopy Science, Hatfield, PA, USA) embedding media at 60°C for 24 hours (according to the manufacturer's protocol). Blocks were trimmed and sectioned using a REICHERT ULTRACUT S ultramicrotome (Leica Microsystems). Semithin sections (1 µm thickness) were stained with 1% toluidine blue in 1% boric acid. Ultrathin sections (70 nm thickness) were collected onto formvar-coated slot grids and counterstained with uranyl acetate and lead citrate. The observation and examination of the sections was performed on a 100kV Jeol JEM1230 (JEOL, Peabody, MA, USA) transmission electron microscope.

Feulgen staining was performed essentially as described.^27^ In brief, eyes were fixed overnight in 4% PFA, dehydrated in an increasing ethanol series, and embedded in Technovit 7100 (Kulzer GmbH, Wehrheim, Germany) according the manufacturer's protocol. Then 3 µm sections were cut with an Autocut 2040 (Reichert, Vienna, Austria), collected on glass slides, and stained by the Feulgen reaction. Slides were examined with a DM5000B microscope (Leica Microsystems) and imaged with a DF490 digital camera using the Leica application suite version 4.8 (Leica Microsystems). Cell types were determined based on position along the apical-to-basal axis and morphology^27^^,^^28^ (apical is toward the retinal pigment epithelium [RPE]). Rod nuclei in the outer nuclear layer (ONL) were distinguished based on their densely stained nuclei, whereas UV cone nuclei were larger, paler, and located basal to the OLM. Elongated red, green, and blue (RGB) cone nuclei are located apical to the OLM.

### Electroretinograms

ERGs were performed essentially as described.^29^ Prerecording preparations were performed under dim red right. Fish were dark-adapted for at least 30 minutes, then anesthetized with tricaine (MESAB; Merck, Darmstadt, Germany). Isolated eyes were positioned on an agarose-filled chamber facing the light source. The recording electrode was filled with E3 (5 mM NaCl; 0.17 mM KCl; 0.33 mM MgSO_4_; 0.33 mM CaCl_2_; pH 7.5 in ddH_2_O) and placed against the center of the cornea, the reference electrode was inserted into the agarose. A stimulus of 100 ms was applied 50 ms after start of tracing. Response to stimulus was recorded three times per sample. Traces were normalized to the baseline, defined by the average potential before the stimulus. The b-wave amplitude was calculated from the trough of the a-wave to the peak of the b-wave.^30^ Results were analyzed using Excel (Microsoft Corp., Redmond, WA, USA), and graphs were prepared in Prism (GraphPad). Smoothing was applied to plotted response curves.

## Results

### Progressive Retinal Vascular Changes Caused by Persistent Hyperglycemia

*Pdx1* mutant zebrafish are a vertebrate genetic model that stably displays key features of human diabetes.^12^ Importantly, homozygous mutants survive to adulthood, thus providing a consistent disease-affected population in which to identify DR phenotypes that progress over time. As DR is clinically characterized in large part based on vascular lesions,^31^ we investigated the retinal vasculature in *pdx1* mutants to determine the presence and extent of DR-associated lesions.

**Figure 1.**
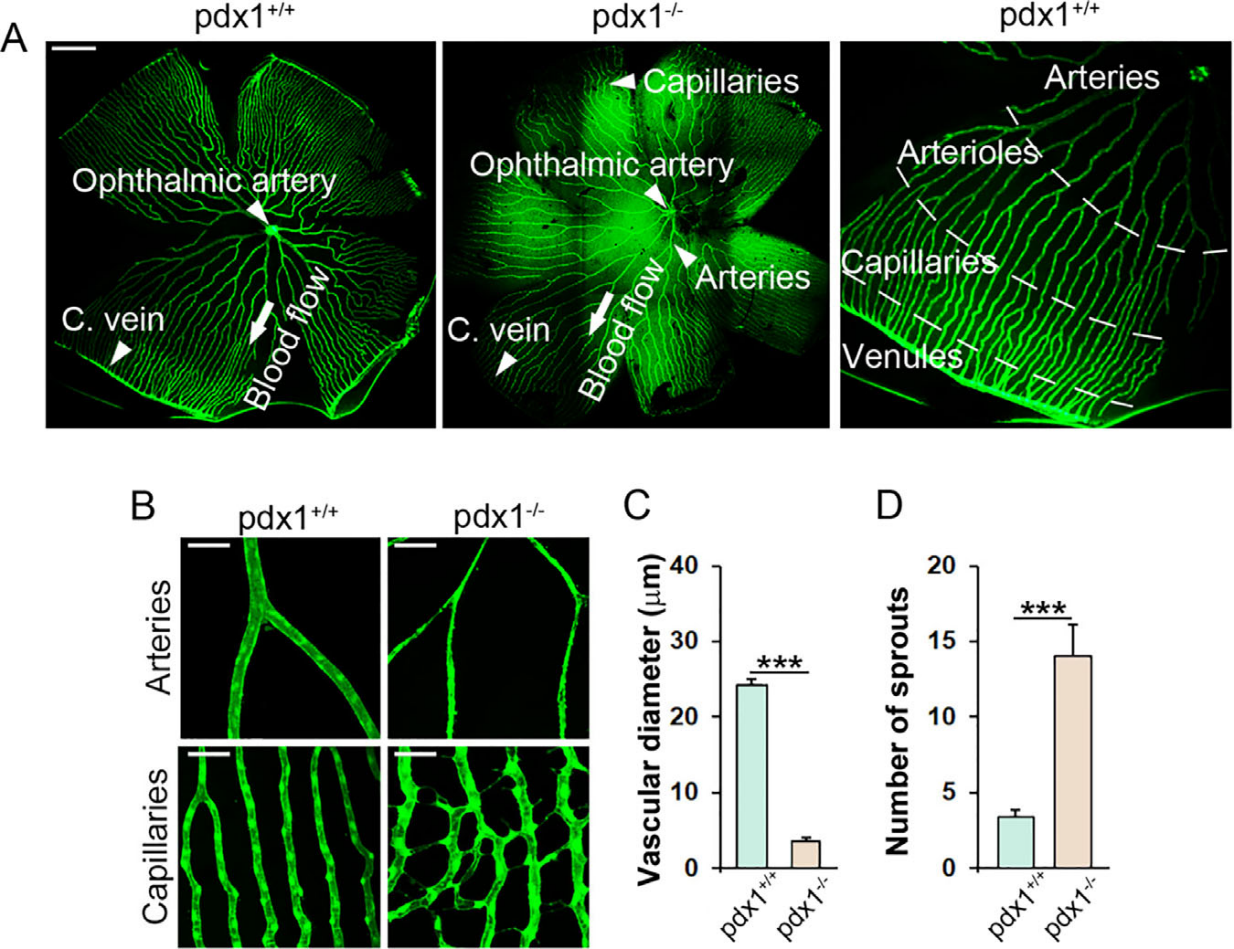
Adult *pdx1* mutants show vascular changes consistent with DR. (**A**) Retina flat mounts from middle-aged (18 months old) *fli1a:EGFP* transgenics, wild type (left) or *pdx1^−/−^* (center), and a close-up view of a wild type (right), with regions of the vascular plexus indicated. Arteries originating from the ophthalmic artery progressively branch to form a peripheral capillary plexus that drains into the circumferential vein (C. vein). Size bar indicates 500 µm. (**B**) Images of retinal vasculature in flat-mounted retinae from middle-aged adult (14–18 month old) *pdx1^+/+^; fli1a:EGFP* controls and *pdx1^−/−^; fli1a:EGFP* mutants focusing on central arteries (top) and peripheral capillaries (bottom). In mutants, arteries show narrowing, whereas capillaries have increased sprouts and branches. Size bars indicate 50 µm. (**C**) Quantitation of vessel diameter in 15 arteries from 4 individual fish per group, and (**D**) sprouting in 10 capillary regions from 3 individual fish per group, from samples as shown in **B**. ***P < 0.001.

The adult zebrafish retina shows a highly organized vascular pattern^13,19^ (Fig. 1A). The retinal vasculature initiates as the ophthalmic artery that pierces the retina and forms a variable number of main artery branches (4–9), which arborize as progressively narrower vessels before forming a capillary plexus. This plexus connects with venous capillaries at the periphery, which drain into the circumferential vein (Fig. 1A).

Vessel morphology was observed in flat mounts of retinae from middle-aged adult (15–18 month) diabetic *pdx1* mutants and age- and genetic background-matched controls expressing the *fli1a:EGFP* transgene. Although global patterning of vessels appeared normal (Fig. 1A), features consistent with DR were detected on closer inspection of the arterial and capillary regions throughout the 360 degrees of the flat-mounted sample. Vessel constriction and points of stenosis were observed in the central arteries (Figs. 1B, top, 1C). Average vessel diameter was reduced seven-fold in *pdx1* mutants. In comparison to controls, retinal capillaries of mutants at this stage showed tortuosity with increased vessel density, sprouting and branching (Fig. 1B, bottom). Sprouts were observed along the length of the capillaries, which in some cases connected with nearby vessels creating a branch (Figs. 1B, bottom, 1D).

Additional vascular lesions were noted in capillaries, as well as arteries, which suggested that cellular integrity was compromised. Specifically, hyperfluorescent and hypofluorescent perivascular deposits were detected adjacent to *fli1a:EGFP*-expressing vessels, suggesting that these contained endothelium-derived EGFP (Supplementary Fig. S1). We hypothesize that these structures, which were not observed in the hypoxia-induced retinopathy model,^19^^,^^20^ could be signs of vascular damage. In zebrafish as in mammals, a specialized choroidal vasculature overlies the RPE. This vessel network, characterized by a sheath-like morphology, appeared unchanged in *pdx1* mutants as compared with controls (Supplementary Fig. S2).

**Figure 2.**
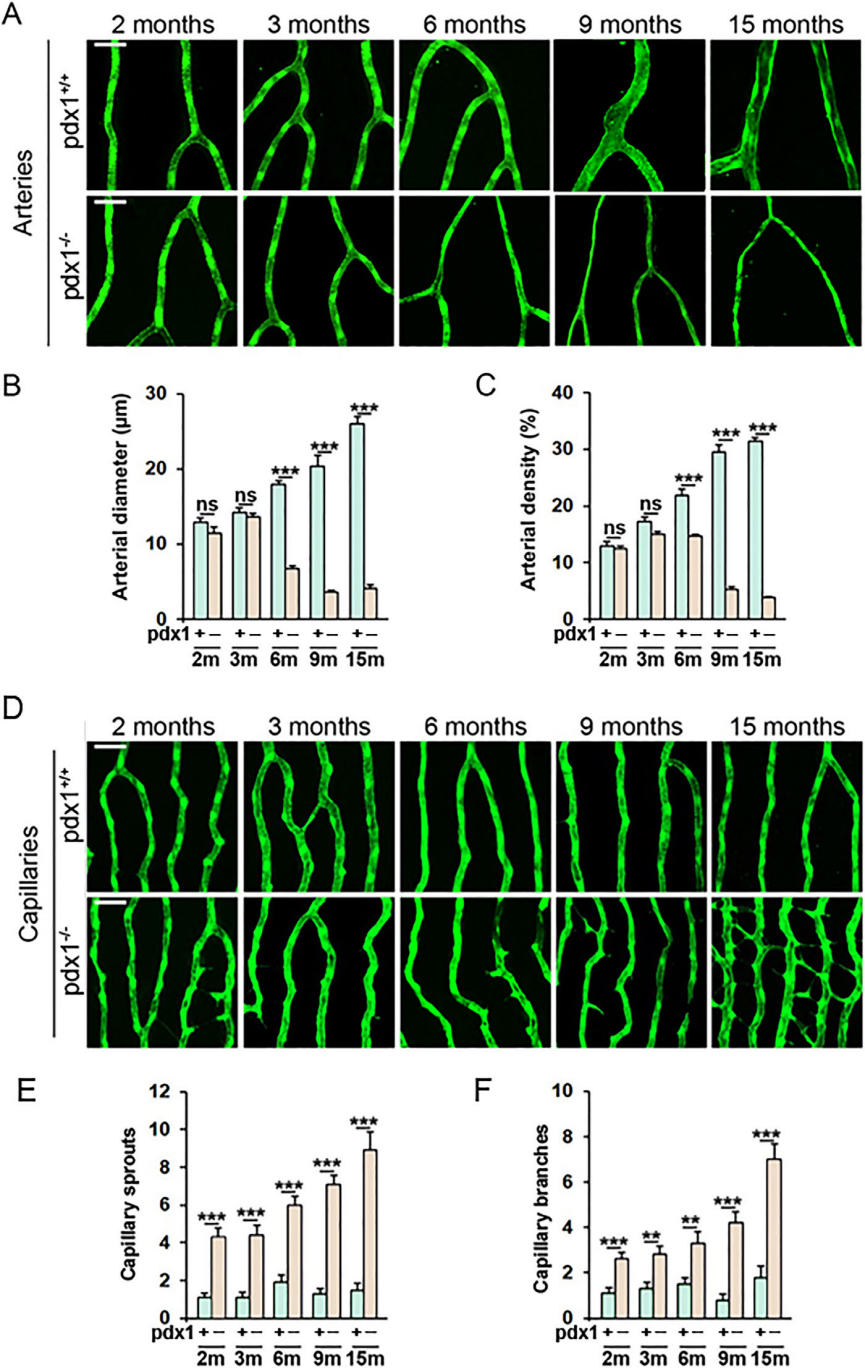
Vascular phenotypes progress over time. Retina flat mounts of *pdx1^−/−^* mutants and *pdx1^+/+^* controls, transgenic for *fli1a:EGFP*, showing representative areas of arteries (**A**) and capillaries (**D**) at the ages indicated, from 2 months through 15 months of age. Size bars indicate 50 µm. (**B**, **C**) Quantitation of arterial diameter (**B**) and density (**C**) in 15 vessels or 15 regions respectively from 4 individual fish per group, from samples as shown in **A**. m, months, ns, not significant. ***P < 0.001. (**E**, **F**) Quantitation of the number of capillary sprouts (**E**) and branches (**F**) in 10 capillary regions from 3 individual fish per group, from samples as shown in **D**. p, *pdx1*, m, months. **P < 0.01, ***P < 0.001.

The hypoxia and VEGF upregulation that are characteristic of DR have been proposed to be a consequence of decreased arterial perfusion, which then triggers capillary sprouting.^32^ To determine the sequence of vascular lesion development in our model, we analyzed retinal vessels at 2, 3, 6, 9, and 15 months in mutants and controls. Significant arterial narrowing and decreased arterial density was detected at 6 months, which progressed in severity at 9 and 15 months (Figs. 2A–C). The capillary phenotype was apparent at earlier stages, with increased sprouts and branching detected already at 2 months. This abnormal vessel growth increased in severity with time, with bursts of increased sprouting observed at 6, 9, and 15 months, correlating with involvement of arterial constriction. At 15 months, the capillaries had developed clear neovascular features (Figs. 2D–F). Overall, these data suggest that early capillary changes may occur prior to, but are likely aggravated by, arterial constriction.

### Molecular Defects in the Vasculature Reflect Disruption of Blood–Retinal Barrier

**Figure 3.**
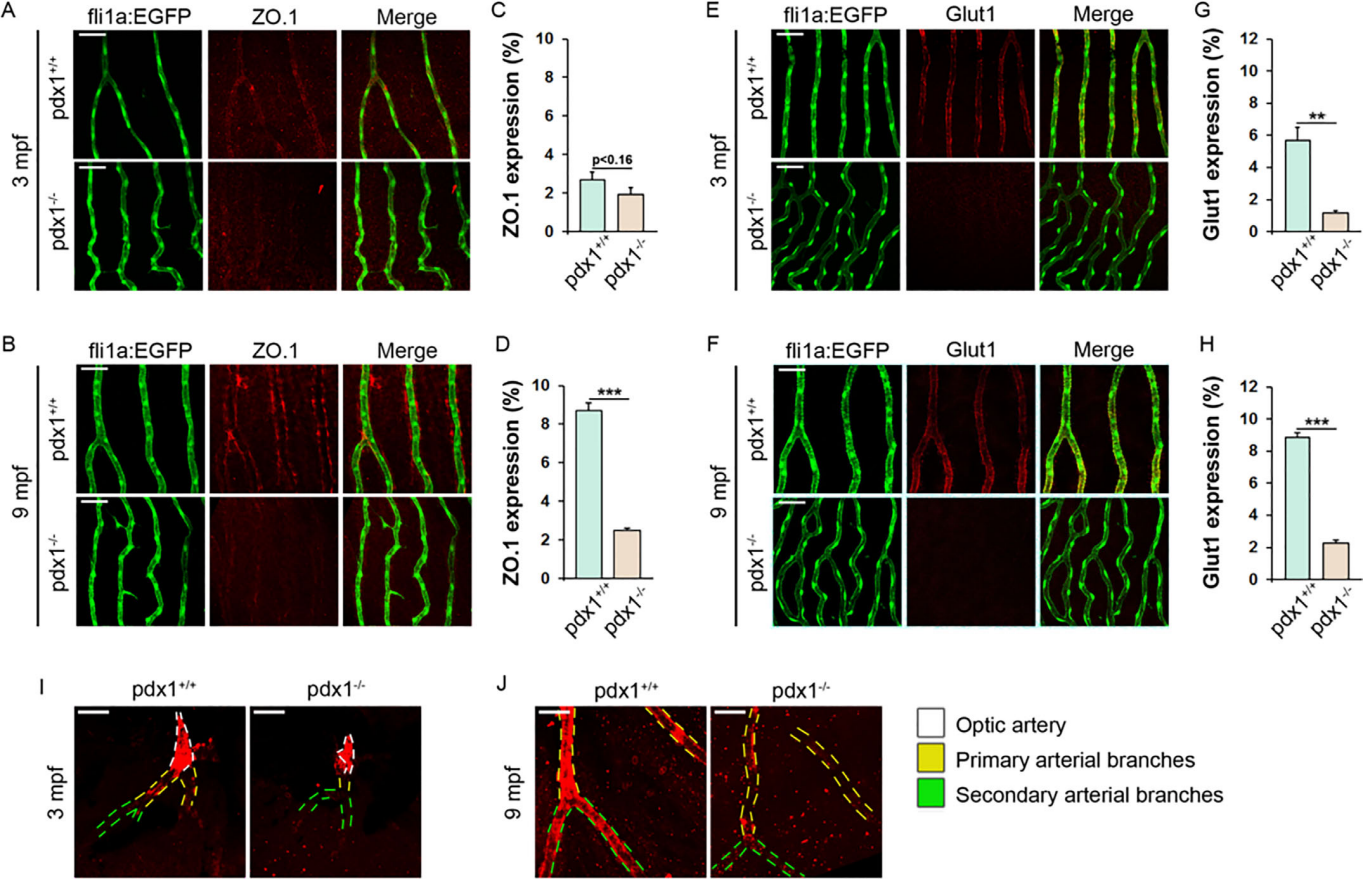
*Pdx1* mutants demonstrate BRB disruption. Retinal flat mounts of 3- and 9-month-old *pdx1* mutants and controls, transgenic for *fli1a:EGFP* and stained with anti-ZO.1 antibody (**A**, **B**), or anti-Glut1 antibody (**E, F**). mpf, months post-fertilization. *fli1a:EGFP* transgene expression (left) and overlap demonstrated in merged images (right). Size bars indicate 50 µm. Quantification of ZO.1- (**C, D**) and Glut1- (**G, H**) expression in retinal vessels, in 10 regions from 3 individual fish per group, from samples as in (**A**, **B**, **E**, **F**). ***P < 0.001, **P < 0.01. (**I, J**) Retinal flat mounts labeled with transgelin1 antibody (red) at 3 mpf (**I**) and 9 mpf (**J**). Arteries are outlined with dashed lines based on the presence of autofluorescent erythrocytes. Optic artery, primary arterial branches, and secondary arterial branches are distinguished by color as indicated. Transgelin1-positive vascular mural cells are associated with primary (yellow dashed lines) or both primary and second order arterial branches (green dashed lines) in 3- and 9-month-old controls, respectively. In 3- and 9-month-old mutants, coverage by transgelin1-positive cells in primary and second-order branches are not observed. Size bars indicate 50 µm.

An intact barrier between the retinal vasculature and parenchyma (blood–retinal barrier [BRB]), formed by endothelial cells, glial cells, and mural cells, maintains tight regulation of nutrient transport, and is essential for retinal integrity and function.^33^ Leaky vessels, which permit influx of signaling mediators and inflammatory cells, are characteristic of DR as endothelial cells become dysfunctional under conditions of metabolic stress.^5^^,^^34^ BRB permeability is regulated through intercellular tight junctions, which contain proteins such as the cytoplasmic adaptor ZO.1.^33^ To define early and progressive changes in BRB integrity in *pdx1* mutants, we examined expression of ZO.1 at 3 and 9 months using immunohistochemistry staining. At 3 months, ZO.1 expression was not yet prominent (Figs. 3A, 3C). The 9 month old controls showed strong ZO.1 expression in perivascular cells and weaker expression in endothelial cells within the capillary network (Fig. 3B, top). By contrast, ZO.1 expression in mutants was strongly reduced at 9 months as compared with controls (Figs. 3B, 3D).

Glut1 is responsible for transport of glucose from the vasculature into the retinal tissue, and is a marker of a mature and healthy BRB.^35^ Changes in retinal Glut1 expression have been reported in DR patients and mouse models, but mechanisms and physiological consequences of this regulation are unclear.^36^ As detected by immunohistochemistry in a flat-mount preparation, Glut1 was expressed in retinal vessels in all regions visualized, particularly in the capillaries (Supplementary Fig. S3). Glut1 expression observed in retinal capillaries of wild types at 3 and 9 months was largely absent in mutants at these stages (Figs. 3E–H).

Loss of pericytes is an early feature of the vascular pathology in DR and contributes to disruption of the BRB by subsequently increasing vascular permeability.^37^ Arterial blood vessels are tightly covered by a layer of vascular mural cells (smooth muscle cells and pericytes), which contributes a supportive scaffold and provides regulatory functions.^23^ Transgelin1, an actin cross-linking protein, is an early marker of mural cells.^23^ At 3 months, transgelin1 expression in mural cells was evident in primary branches of the optic artery in controls but not in mutants (Fig. 3I). At 9 months, both primary and secondary arterial branches were covered with transgelin1-positive mural cells in controls, whereas only primary branches were associated with such cells in the mutants (Fig. 3J). In middle-aged controls (18 months), tertiary arteriolar branches were enriched in transgelin1-positive mural cells, whereas only primary arterial branches of the optic artery were covered by these cells in the mutants (Supplementary Fig. S4).

**Figure 4.**
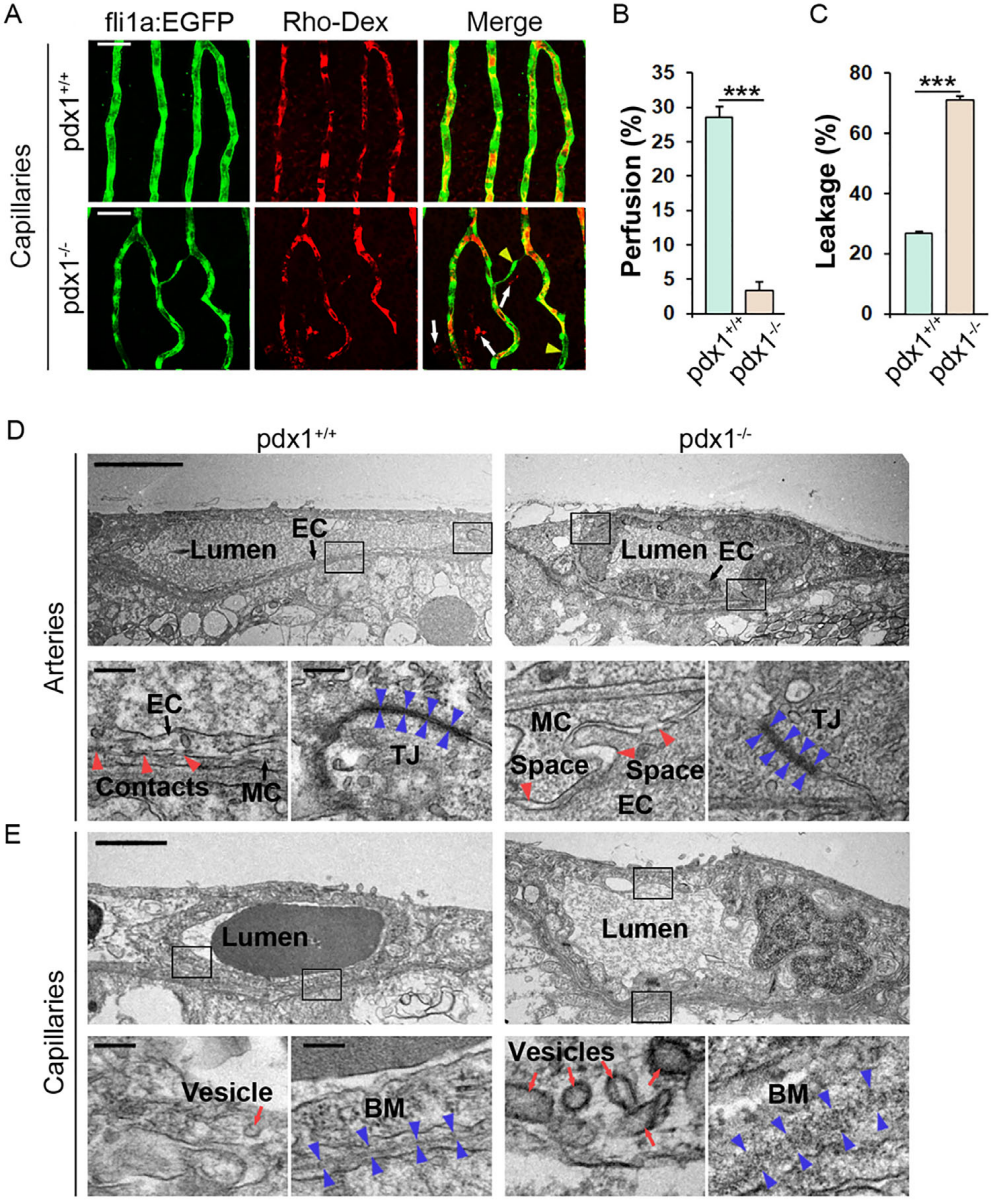
(**A**) Retinal flat mounts from 6-month-old *pdx1* mutants and controls, transgenic for *fli1a:EGFP* and previously injected with rhodamine-labeled dextran (Rho-Dex, red). Areas of poor Rho-Dex perfusion through the vessels (yellow arrowheads) and areas of Rho-Dex leakage (white arrows) are clearly evident in *pdx1* mutants but not in controls. Size bars indicate 50 µm. (**B**, **C**) Quantification of perfusion (**B**) and leakage (**C**) in the retinal capillaries in 10 regions of 3 individual fish per group from samples as shown in **A**. ***P < 0.001. (**D, E**) TEM images of retinal sections from 6-month-old *pdx1* mutants and controls focusing on arterial (**D**) or capillary (**E**) vessels, showing swollen endothelium, loosened contacts between endothelial cells (EC), and mural cells (MC, red arrowheads), and partially dissolved, loosened tight junctions (TJ, blue arrowheads) in the arteries of mutants compared with controls (**D**). Also shown is an increased number of vesicles (red arrows), and disrupted basement membrane (BM) in the capillaries of mutants compared with controls (**E**). Black boxes in the overview images (upper rows) indicate the areas magnified in the images below (lower rows). Size bars in overview and magnified images indicate 5 µm and 100 nm, respectively.

Vessel leakiness may be a consequence of pathological disruption of the BRB, but is also characteristic of newly formed vessels.^4^ To directly demonstrate vessel leakage, retinae were harvested from 6-month-old *fli1a:EGFP* transgenic fish 90 minutes following intraperitoneal injection of low molecular weight (70kD) rhodamine dextran (Rho-Dex). Examination of vessels in flat mounts revealed extravascular exudates in *pdx1* mutants, that were absent in control samples (Figs. 4A, 4C). These exudates were found adjacent to established vessels, as well as in regions of neovascular branches and sprouts. This suggests leakage from both breakdown of previously functional barriers and from insufficient barrier formation in newly sprouting vessels. We also quantitated the degree to which the injected Rho-Dex filled the capillary vascular space, defined by *fli1a:EGFP* transgene expression, as an indication of vascular perfusion. This analysis revealed significantly reduced vessel perfusion in mutants as compared with controls (Fig. 4B).

To define early changes at the ultrastructural level, we examined retinal vessels at 6 months using transmission electron microscopy (TEM). Retinal arteries in wild types were as expected: thin-walled, exhibited highly condensed tight junctions, and established close contacts with vascular mural cells (Fig. 4D, left). In mutant fish, however, the endothelium was dramatically thickened and swollen, likely causing the observed luminal constriction and hypoperfusion observed from 6 months onward (Fig. 4D, right). Furthermore, tight junctions appeared less dense, with wider interendothelial space within the junctions compared with age-matched controls. Mural cell coverage was less tight and fluid-filled spaces were frequently found between endothelial and vascular mural cells of the mutant fish (Fig. 4D, right).

In the capillary region, control fish exhibited relatively thin endothelia, albeit with a few vesicles likely important for transport across the BRB (Fig. 4E). The basement membrane was condensed and continuous in this region. In the mutants, however, the endothelium was thickened and contained many more vesicles, including several macropinosomes that were not observed in control fish, suggesting elevated transendothelial transport and leakage of fluid. The basement membrane was much less condensed and appeared discontinuous and partly degraded (Fig. 4E). Overall, these findings are consistent with disruption of the BRB, with increased vessel permeability and leakiness, in the retina of *pdx1* mutants.

### Neural-Retina Changes in *pdx1* Mutants Affect Photoreceptors and Müller Glial Cells

**Figure 5.**
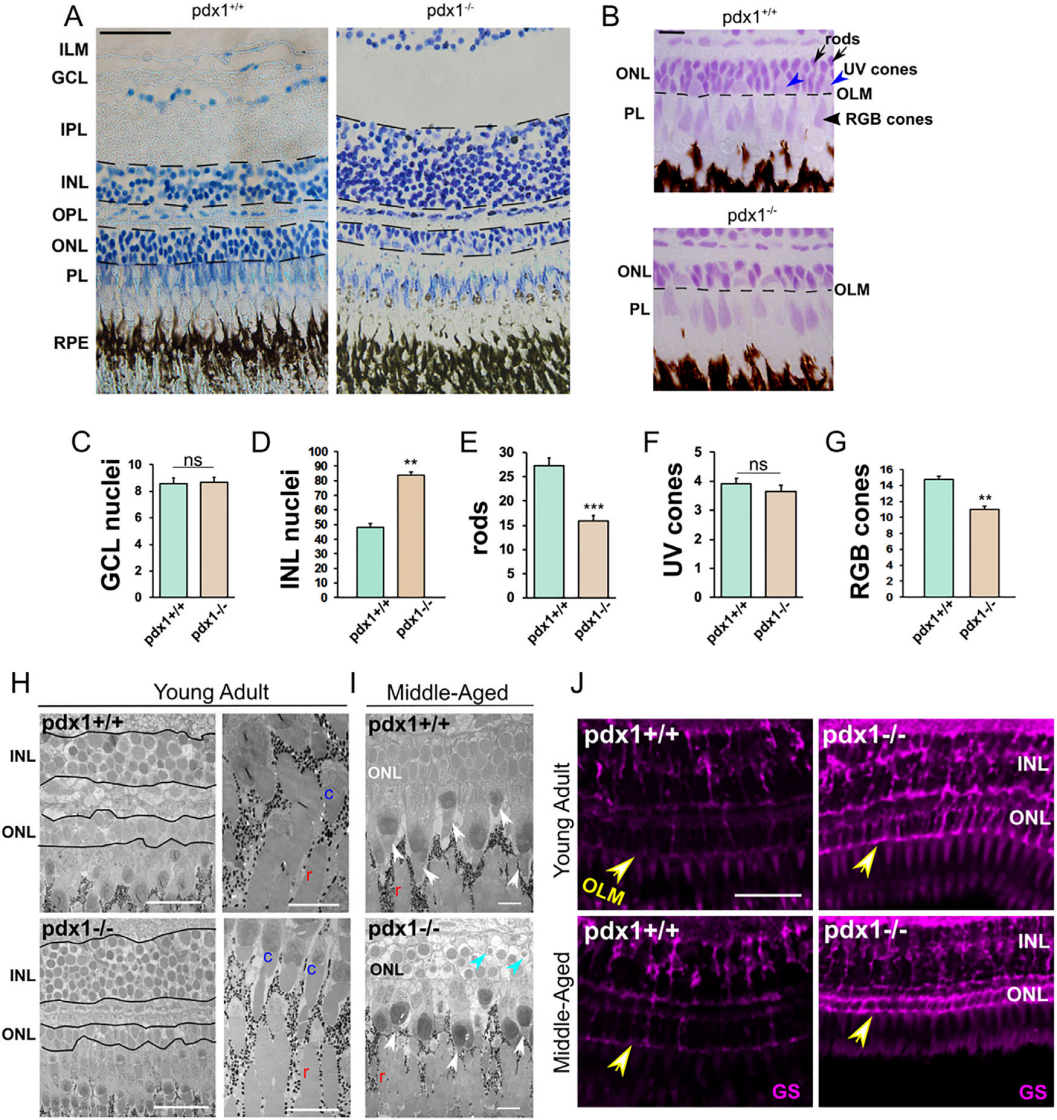
Pathology in the neural-retina of *pdx1^−/−^* mutants. Toluidine blue (**A**) and Feulgen (**B**) staining in sections of the outer retina from middle-aged (12 month) *pdx1* mutant or control zebrafish. Based on Feulgen histology, rod photoreceptor nuclei in the ONL are densely stained (**B**, top, black arrows), whereas UV cone nuclei located apically within the ONL are larger and lighter stained (**B**, top, blue arrowheads). Elongated nuclei of RGB cones are found apical to the OLM (**B**, top, black arrowhead). Size bar indicates 50 µm (**A**) or 10 µm (**B**). (ILM, inner limiting membrane; GCL, ganglion cell layer; IPL, inner plexiform layer; INL, inner nuclear layer; OPL, outer plexiform layer; ONL, outer nuclear layer; PL, photoreceptor layer; RPE, retinal pigment epithelium; OLM, outer limiting membrane). **C**-**G** Quantification of indicated cell types (for details, see Methods section) in 10 or more regions of 50 µm width from 3 individual fish per group (from samples as shown in **A**, **B**). **P < 0.001, ***P < 0.0001, ns, not significant. (**H**, **I**) TEM images of the outer retina from 6-month-old young (**H**) or middle-aged adult (14–16 months) (**I**) controls and *pdx1* mutants. Similar to middle-aged samples, young adult mutants show reduced ONL (**H**, left). Rod (r) and cone (c) outer segments can be identified. In middle-aged adults, cone outer segments (**I**, white arrowheads) appear shortened in mutants as compared with controls. In addition, pyknotic nuclei (blue arrowheads) are present in the ONL of *pdx1* mutants (**I**, bottom) (c, cone, r, rod). Size bars indicate 25 µm (**H**, right), 5 µm (**H**, left), or 10 µm (**I**). (**J**) Cryosections of controls (left) and *pdx1^−/−^* mutants (right) at young adult (7 months) and middle age (17 months), immunostained with anti-glutamine synthetase (GS, magenta). *Pdx1^−/−^* mutants show enhanced signal in the outer retina, and hypertrophic processes at the OLM (arrow). (*pdx1^+/+^*, n = 4; *pdx1^−/−^*, n = 6). Size bar indicates 20 µm.

Pathological changes in retinal vessels, visualized by ophthalmoscope examination, have historically been the major criteria for assessing DR in patients.^2^^,^^38^ However, vessel alterations are often accompanied by alterations in retinal neurons that are more challenging to detect.^38^ The zebrafish retina, comparable to all vertebrates including mammals, contains three nuclear and two synaptic layers. Neuronal cell bodies are found in nuclear layers, whereas axonal and dendritic processes extend and make connections within the intervening plexiform layers. The INL contains the cell bodies of neural bipolar, horizontal, and amacrine cells, as well as the Müller glial cells. Photoreceptors reside in the ONL and adjacent photoreceptor layer, their light-collecting outer segments are supported by processes from the underlying RPE (Fig. 5A).

Histological examination of retinal sections from middle-aged controls and *pdx1* mutants (12 months old) revealed differences in the outer and inner nuclear layers (Figs. 5A). Mutants showed increased nuclei in the INL and decreased nuclei in the ONL (Figs. 5A, 5B, 5D). Closer analysis of photoreceptors, distinguished based on nuclear morphology and location,^28^^,^^39^ revealed that rods and RGB cones were significantly reduced (Figs. 5E, 5G), whereas UV cone numbers were essentially unchanged in mutants as compared with controls (Fig. 5F). Cell numbers in the ganglion cell layer, which contains the output neurons of the retina, were comparable in mutants and controls (Fig. 5C). We additionally analyzed retinae from young (6 month) and middle-aged (15–16 months) adults by TEM. Changes in ONL and INL were already present in young adult samples (Fig. 5H). Middle-aged adults additionally demonstrated pyknotic nuclei in the ONL, and shortened cone outer segments (Fig. 5I).

Müller glial cells of the retina reinforce retinal architecture and help maintain metabolic homeostasis of retinal neurons.^40^ To aid in this supportive role, processes of Müller glia cells extend laterally within the retina, and span radially to reach the outer limiting membrane.^41^ Following injury in the retina, Müller glial cells become activated and show hypertrophy in a process referred to as reactive gliosis.^41^^,^^42^ To examine the status of Müller glial cells in *pdx1* mutants, we performed antibody staining for the specific marker GS. Hypertrophic changes were observed starting from young adult stages, with enhanced expression of GS (Fig. 5J, Supplementary Fig. S5). Specifically, we could clearly observe Müller cell processes encircling nuclei in the ONL (Fig. 5J, Supplementary Fig. S5A), as has been previously described following photolesion damage to zebrafish retina.^42^ These changes were observed at stages spanning young adult to middle age, suggesting persistent activation.

### Photoreceptor Deficiencies Correlate with Dysfunction of the Outer Retina

**Figure 6.**
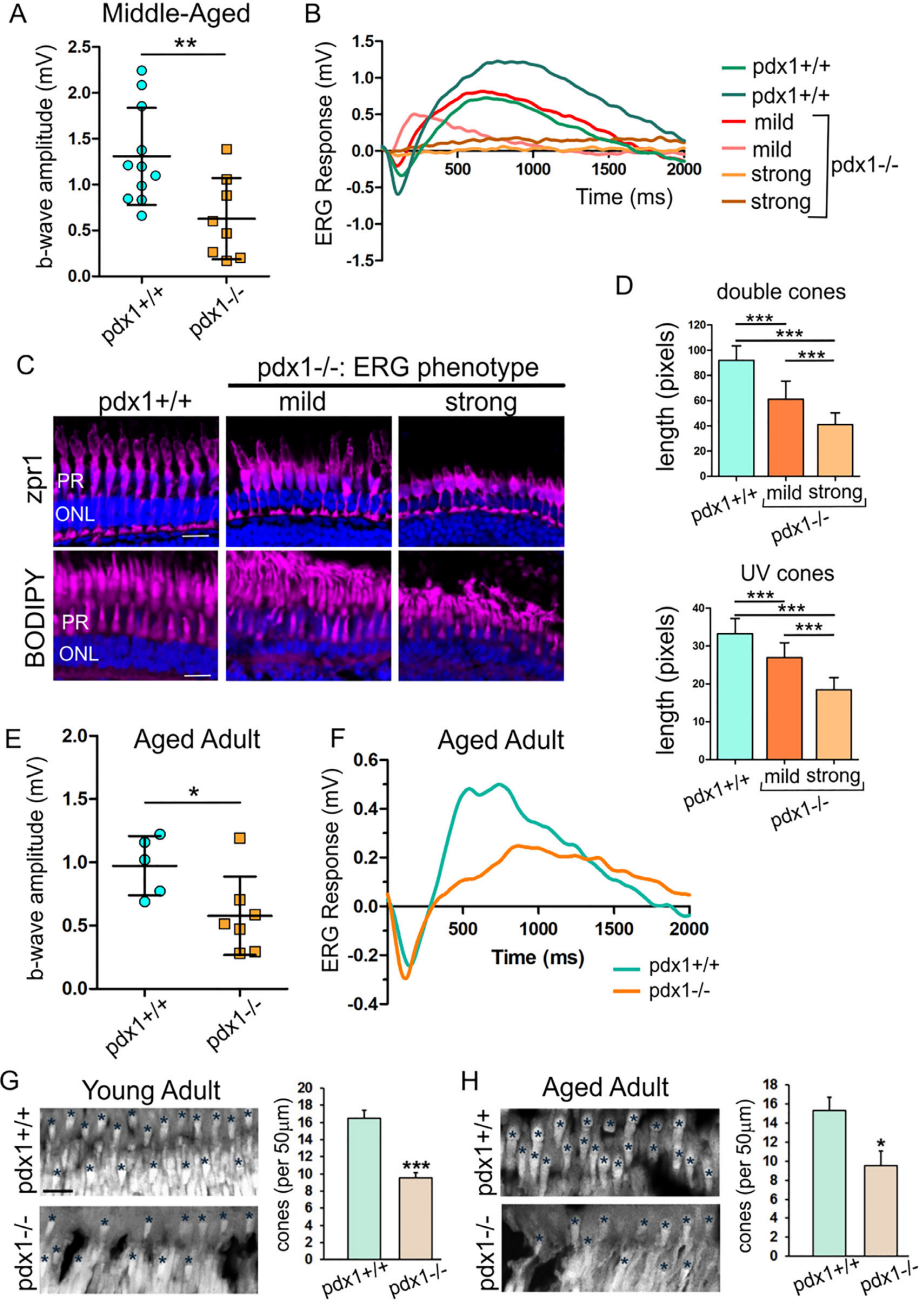
Retinal functional defects and photoreceptor loss are detected in *pdx1^−/−^* mutants. (**A**) b-wave amplitude of wild type and *pdx1^−/−^* mutants middle-aged adults (11 months old), as measured by ERG (**A**, *pdx1^+/+^*, n = 11; *pdx1^−/−^*, n = 8, **P < 0.01, unpaired *t*-test). (**B**) ERG recordings from representative controls, mutants with no to mild defect in response (mild) and with strong b-wave reduction phenotype (strong). (**C**) Cryosections labeled with anti-Zpr1 (top, magenta) and BODIPY (bottom, magenta), with nuclei counterstained by DAPI (blue), from samples with ERG tracings as shown in **B (**control, n = 2; mild, n = 2; strong, n = 2). Size bars indicate 10 µm. (**D**) Quantitation of length of double cones (top) and UV cones (bottom), measured from images as shown in (**C).** (Details of quantitation are described in the Methods section.) (**E**) b-wave amplitudes from ERG recordings of aged adults (*pdx1^+/+^*, n = 5; *pdx1^−/−^*, n = 7, *P < 0.05, unpaired *t*-test). (**F**) Electroretinogram recordings from representative aged adult (>24 months old) *pdx1^+/+^* control (green) and *pdx1^−/−^* mutant (orange). (**G, H**) Sections of the outer nuclear layer and photoreceptors from young (**G**) or aged adult (**H**) *pdx1* mutants or controls stained with DAPI (gray). Graph indicates quantification of the number of hyperfluorescent cones in 5 regions of 50 µm in width from 3 individual young adult (7 months) (**G**) or aged adult (>24 months) (**H**) samples as shown. *P < 0.05, ***P < 0.001, unpaired *t*-test. Size bar indicates 20 µm.

We next determined whether the changes in the neural retina in *pdx1* mutants caused corresponding defects in retinal function. An ERG, which detects light-induced field potential change in the whole retina, can be used to analyze outer retinal function.^43^ We measured ERG responses to bright light stimuli in dark-adapted middle-aged (11 months old) adults, which provides a larger amplitude response compared with light-adapted testing, and interrogates function of both rods and cones.^44^ Specifically, the b-wave amplitude represents the depolarization of on-bipolar cells induced by the hyperpolarization of photoreceptors. Reduced b-wave amplitude reflects disruption of synaptic transmission downstream of photoreceptors. Consistent with perturbation of photoreceptor function, average b-wave amplitude was reduced in the mutants (Fig. 6A, **P < 0.01).

To correlate ERG responses with the status of photoreceptors, we labeled retinal sections with BODIPY to highlight lipid membranes, and Zpr1 antibody to label red-green double cones.^45^ We examined retinae from controls, mutants that showed mild or no defect in ERG response (mild), and mutants that showed a strong b-wave reduction phenotype (strong, Fig. 6B). In controls, double cones have an elongated columnar morphology revealed by Zpr1 antibody staining (Fig. 6C). Zpr1 staining in retinae from mutants with mild ERG phenotype appeared less regular and cones showed significant shortening (Figs. 6C, 6D, top). In mutants with strongly reduced b-wave amplitude, these cones had a rounded shape and the length was further decreased. Based on their cell body location in the ONL, BODIPY-labeled UV cone morphology can be quantitated.^24^^,^^26^ Although by histology we found the number of UV cones to be unchanged (Fig. 5F), BODIPY labeling revealed disorganized and shortened UV cones in mutants, with the severity correlating with reduced ERG response (Figs. 6C, 6D, bottom). Overall, this analysis shows that photoreceptors were impacted at both the morphologic and functional level.

As DR leads to blindness in human patients, we examined whether visual deficits increased in severity during the aging of the mutants. Although *pdx1* mutants show decreased viability as adults,^12^ rare mutants survive into later adulthood. In aged adults (>24 months post-fertilization), ERGs of mutants showed b-wave amplitudes that were significantly reduced compared with controls (Fig. 6E, *P < 0.05, 6F). However, they were not completely lacking in response. Interestingly, the mean ERG response in mutants between middle-aged (b-wave amplitude = 0.63 ± 0.44 mV) and aged adult (b-wave amplitude = 0.58 ± 0.31 mV) was similar (*P* = 0.99, unpaired *t*-test). To determine changes in overall cone status over time, we quantified cone numbers based on DAPI staining of the outer segments.^46^ In young adult, as well as in aged adults, comparing mutant to age- and background-matched controls, a significant decrease in cone photoreceptor number was detected (Figs. 6G–H). Thus although there was a detectable and significant defect in retinal function and a correlating pathology in photoreceptors, it was not more severe in mutants that survived for more than 2 years.

## Discussion

We describe here the *pdx1* mutant zebrafish^12^ as a new and clinically relevant model for DR. In contrast to previous models limited to short duration treatments and assessing phenotypes at a single time point, we report a DR model amenable to being assessed at early as well as later time points. We observed progressive retinal vascular pathologies affecting both arteries and capillaries, as well as degenerative changes in photoreceptors.

Recent mouse DR models feature multiple genetic alterations, or take advantage of variation in background genetic susceptibilities, in attempts to better represent human disease. However, these models often do not represent genetic modifications associated with human disease,^47^ or are so severely hyperglycemic as to only permit development of early pathologies.^48^ In addition to type 1 diabetes models, manipulations of rodents that recapitulate the metabolic disturbances of type 2 diabetes show evidence of retinal neurologic and vascular disruptions,^9^^,^^49^ but manifest mainly the early stages of disease development as seen in humans.

The *pdx1* mutant zebrafish most resembles human neonatal diabetes, as the early onset diabetic state occurs only in homozygous mutants and is associated with exocrine pancreas developmental defects.^12^^,^^50^ *Pdx1* mutations are a rare cause of diabetes in the human population, with clinical manifestations that vary depending on the precise genetic lesion. Although our model is based on a rare condition in humans, it is valuable in creating a β-cell deficient and hyperglycemic state that recapitulates the persistent metabolic dysregulation of human diabetes. Severity of human DR, independent of diabetes etiology, is foremost associated with duration of diabetes and degree of hyperglycemia.^4^ Future studies in the *pdx1* mutant zebrafish model can explore how gender, additional genetic loci, and environmental factors such as diet, modify DR onset and progression.

In contrast to glucose-induced DR in zebrafish, in which cones were found to be more susceptible to disruption,^16^ our model shows perturbation affecting both rods and cones. Several factors could account for this difference, in particular the nature of the initiating treatment and the persistence of disease state. Zebrafish diabetes models of limited duration^16^^,^^51^ may elicit modest downstream pathologies as compared with effects seen with sustained genetic disruption of glucose homeostasis, as in the *pdx1* mutant.

In rodent DR studies, there is loss of photoreceptors and degeneration of photoreceptor outer segments, variably impacting rods and/or cones, depending on the diabetes model.^52^ Photoreceptor changes in diabetic patients have not yet been widely assessed, but several studies have shown pathologic effects.^52^ Candidate factors for mediating tissue damage in DR include reactive oxygen species (ROS), hypoxia, and immune system activation, although the precise molecular sequence of events remains unclear.^34^ Photoreceptors are the most metabolically active cells in the retina, with the consequence of high mitochondrial activity and increased ROS generation.^53^ Rod and cone outer segments, cellular compartments with minimal antioxidant activity, undergo continuous removal and renewal to replace damaged cellular components and maintain high visual function.^54^ One hypothesis is that, in retinal disease states, insufficient renewal of damaged outer segments compromises photoreceptor viability.^54^

Vascular development and vascular growth in response to hypoxia are well studied, and zebrafish has been a fruitful model in these investigations.^20^ Although inappropriate new vessel formation (neovascularization) leads to devastating vision loss in DR, the initiating lesions are characterized by vessel drop-out.^55^ Because of a lack of good models to study these first events, few molecular players have been identified, resulting in limited therapeutic options to counteract vessel loss in early DR. The zebrafish DR model we report here demonstrated characteristic early vascular changes, including pericyte loss, BRB breakdown, and decreased perfusion, and further investigations will aim to discover responsible signaling pathways.

*Pdx1* diabetic mutants showed consistently progressing vascular changes that at later stages highly correlated with human PDR. By contrast, we did not see relentless progression in the retinal neuron phenotype. We did observe persistent upregulation of GS, a Müller glial protein that serves as a potent neuroprotectant.^56^ This response could serve to promote photoreceptor survival in the face of toxic metabolic injury, and help to maintain visual function, even with advanced age. Future studies will address whether, in our metabolic retinopathy model, Müller glial activation serves primarily a neuroprotective function, or whether there is an accompanying regenerative response, as seen in other zebrafish retinal injury models.^41^

## Conclusions

Overall, this study demonstrates that *pdx1* mutant zebrafish manifest vascular, neuronal, and functional attributes of DR. The possibility to apply noninvasive visualization of the retina and vasculature,^57^ in combination with in vivo vision assessment,^43^^,^^58^ will enable correlation of pathophysiology and progression of both vascular and retinal phenotypes. Our long-duration DR model offers the opportunity to study injury responses downstream of pathologic metabolic stress in the retina, which can reveal new targets for therapeutic modulation.
