# Supplementary material for: Photoreceptor Degeneration Accompanies Vascular Changes in a Zebrafish Model of Diabetic Retinopathy
Source: Invest Ophthalmol Vis Sci. 2020 Feb 27;61(2):43. doi: 10.1167/iovs.61.2.43 (PMC7329949; doi:10.1167/iovs.61.2.43)
Supplement: Supplementary file 1 [file iovs-61-2-43_s001.pdf]

Supplemental Figures

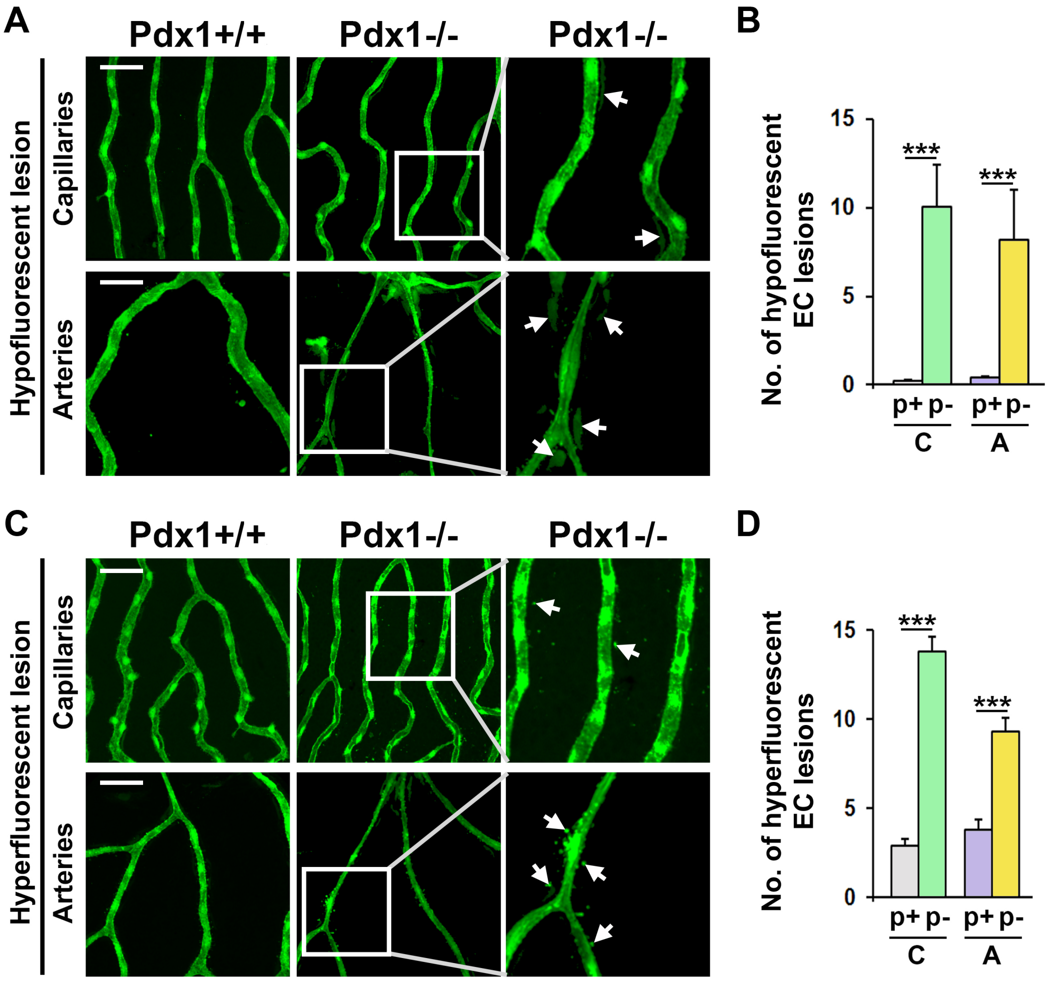

Figure S1. *Pdx1* mutant zebrafish develop vascular lesions in the retina. (A, C) Confocal micrographs of retinal flat mounts from *pdx1* mutant or controls, transgenic for *fli1a:EGFP*. Hypofluorescent (A) and hyperfluorescent (C) abluminal accumulations of shedded material from the endothelium (lesions, white arrows) are apparent in the mutants but not in the controls. Size bars indicate 50 μm. (B, D) Quantification of the number of hypo- (B) or hyper-fluorescent (D) lesions in 10 regions from three individual fish per group from the experiment shown in (A, C). ***:$p<0.001$
