# Supplementary material for: Photoreceptor Degeneration Accompanies Vascular Changes in a Zebrafish Model of Diabetic Retinopathy
Source: Invest Ophthalmol Vis Sci. 2020 Feb 27;61(2):43. doi: 10.1167/iovs.61.2.43 (PMC7329949; doi:10.1167/iovs.61.2.43)
Supplement: Supplementary file 2 [file iovs-61-2-43_s002.pdf]

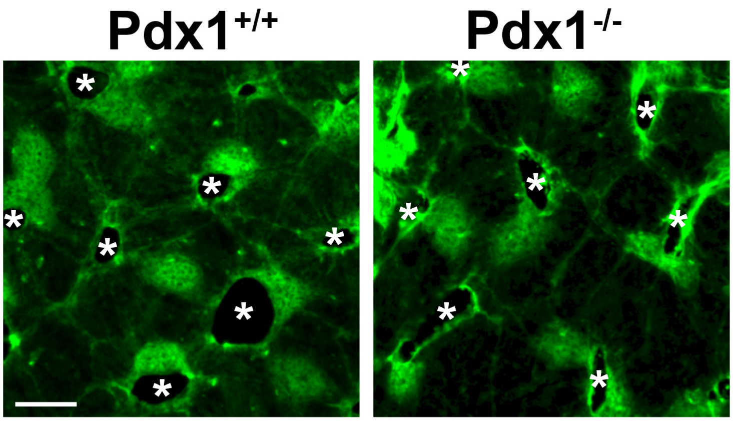

Figure S2. The choriocapillaris is not changed in *pdx1* mutants. Confocal micrographs of choriocapillaris flat mounts from middle-aged (12 month old) *pdx1* mutant or control zebrafish transgenic for *fli1a:EGFP* (green). White asterisks indicate the non-vascular transluminal interstitial pillars.
