# Supplementary material for: Photoreceptor Degeneration Accompanies Vascular Changes in a Zebrafish Model of Diabetic Retinopathy
Source: Invest Ophthalmol Vis Sci. 2020 Feb 27;61(2):43. doi: 10.1167/iovs.61.2.43 (PMC7329949; doi:10.1167/iovs.61.2.43)
Supplement: Supplementary file 3 [file iovs-61-2-43_s003.pdf]

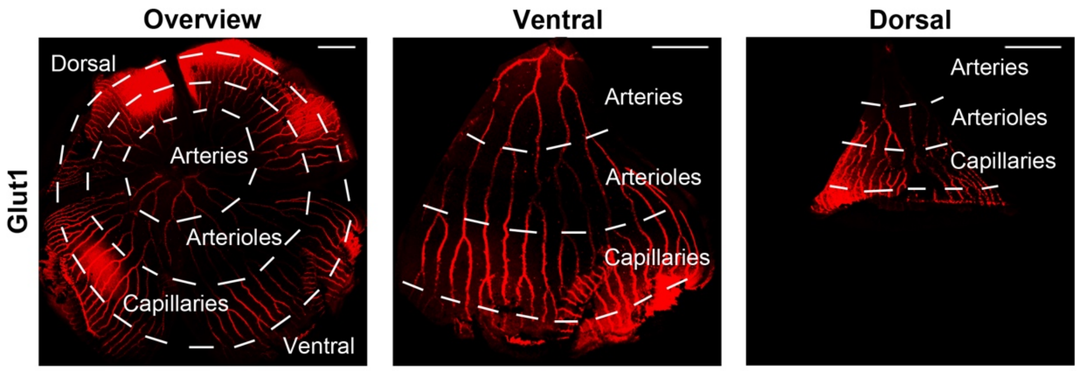

Figure S3. Glut1 expression in retinal vessels. Retinal flat mount of a wild type middle-aged adult (18 month old) immunostained for Glut1 (red). Dorsal and ventral areas, and the regions occupied by arteries, arterioles and capillaries, are indicated. Glut1 expression is prominent in capillaries of both dorsal and ventral regions. Size bars indicate 500 μm.
