# Supplementary material for: Photoreceptor Degeneration Accompanies Vascular Changes in a Zebrafish Model of Diabetic Retinopathy
Source: Invest Ophthalmol Vis Sci. 2020 Feb 27;61(2):43. doi: 10.1167/iovs.61.2.43 (PMC7329949; doi:10.1167/iovs.61.2.43)
Supplement: Supplementary file 4 [file iovs-61-2-43_s004.pdf]

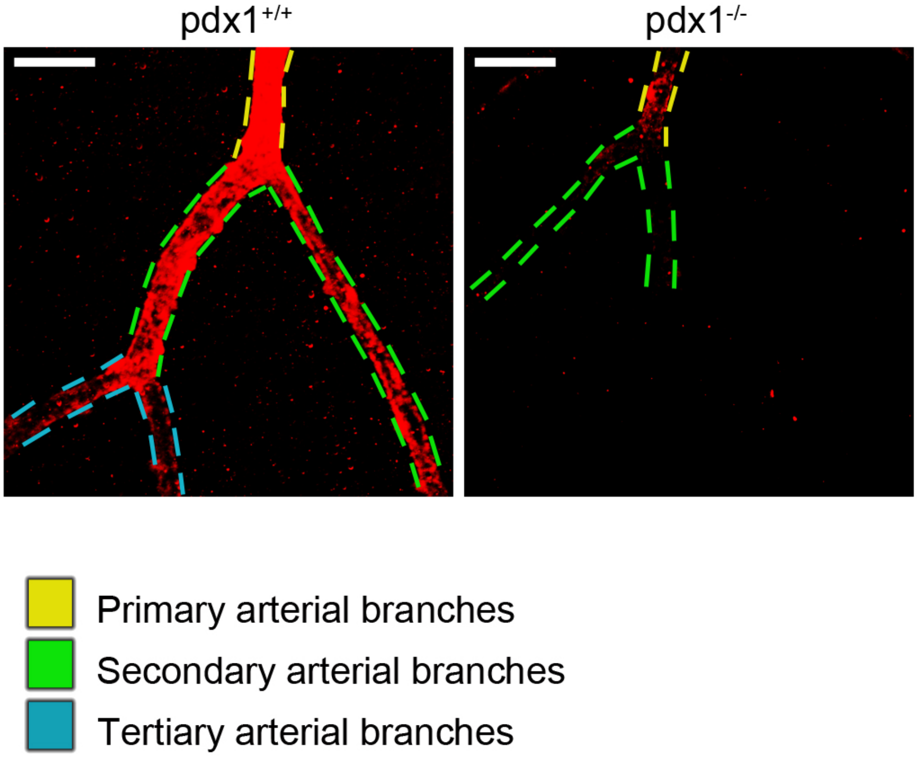

Figure S4. Expression of Trangelin1 in vascular mural cells. Retinal flat mounts of middle-aged (18 months old) wild type and *pdx1-/-* zebrafish stained for Transgelin1 expression (red). Primary arterial branches, secondary arterial branches and tertiary arterial branches are outlined with dashed lines in different colors as indicated. Size bars indicate 50 μm.
