# Supplementary material for: Photoreceptor Degeneration Accompanies Vascular Changes in a Zebrafish Model of Diabetic Retinopathy
Source: Invest Ophthalmol Vis Sci. 2020 Feb 27;61(2):43. doi: 10.1167/iovs.61.2.43 (PMC7329949; doi:10.1167/iovs.61.2.43)
Supplement: Supplementary file 5 [file iovs-61-2-43_s005.pdf]

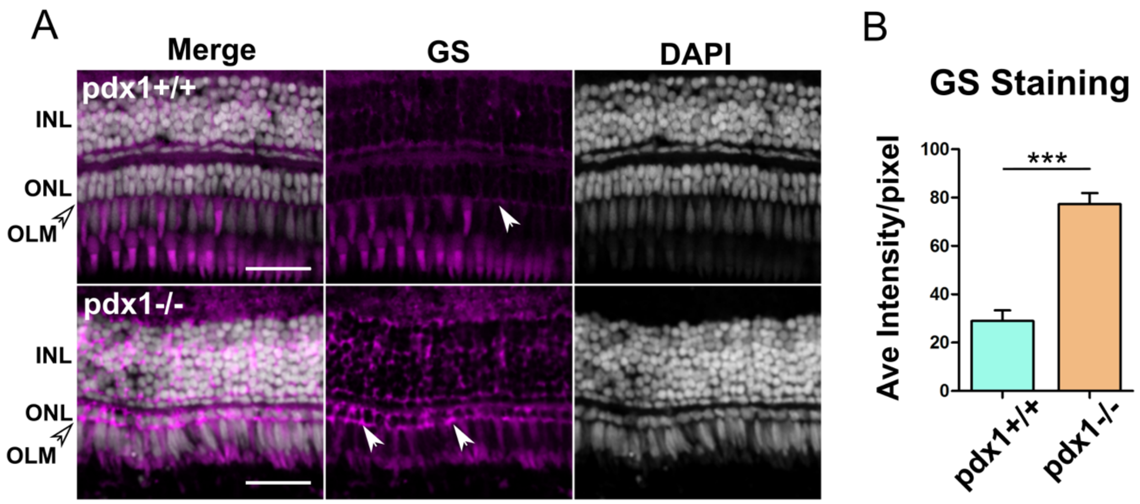

Figure S5. Upregulation of Glutamine Synthetase in the retinas of *pdx1-/-* mutants. A. Cryosections of middle-aged (11 months old) controls (top) and *pdx1-/-* mutants (bottom), immunostained with anti-glutamine synthetase (magenta), nuclei are labeled by DAPI (gray). Hypertrophic processes are observed at the OLM (arrowheads) in *pdx1-/-* mutants. (INL: inner nuclear layer, ONL: outer nuclear layer, OLM: outer limiting membrane). Size bars indicate 20 μm. B. Graph depicting GS staining intensity in *pdx1+/+* compared to *pdx1-/-* mutants, analyzed from samples as in (A) and Fig. 5J. *pdx1+/+*, n=11 regions from 4 samples; *pdx1-/-*, n=20 regions from 6 samples. Error bars represent SEM. ***, p<0001.
